# Influence of Different Framework Designs on the Fracture Properties of Ceria-Stabilized Tetragonal Zirconia/Alumina-Based All-Ceramic Crowns

**DOI:** 10.3390/ma9050339

**Published:** 2016-05-05

**Authors:** Tomofumi Sawada, Sebastian Spintzyk, Christine Schille, Ernst Schweizer, Lutz Scheideler, Jürgen Geis-Gerstorfer

**Affiliations:** Section Medical Materials Science & Technology, University Hospital Tübingen, Osianderstrasse 2-8, Tübingen 72076, Germany; Sebastian.Spintzyk@med.uni-tuebingen.de (S.S.); christine.schille@med.uni-tuebingen.de (C.S.); ernst.schweizer@med.uni-tuebingen.de (E.S.); lutz.scheideler@med.uni-uebingen.de (L.S.); geis-gerstorfer@mwt-tuebingen.de (J.G.-G.)

**Keywords:** ceria-stabilized tetragonal zirconia/alumina, framework modification, all-ceramic restoration, zirconia, chipping, porcelain veneer

## Abstract

The aim of this study was to evaluate the fracture load and failure mode of all-ceramic crowns with different ceria-stabilized tetragonal zirconia/alumina nanocomposite (Ce-TZP/A) framework designs. Four frameworks (anatomical shape: AS, with a buccal or lingual supporting structure: BS and LS, or buccal and lingual supporting structures: BLS) were fabricated. All frameworks were veneered with porcelain to fabricate all-ceramic crowns followed by cementation to tooth analogs. The fracture load of each crown either without or with pre-loading (1.2 million cycles, 49 N) was measured. The failure mode was classified into partial or complete fracture. Differences were tested for significance (*p* < 0.05) by a two-way Analysis of Variance (ANOVA), followed by Tukey’s test and by Fisher’s exact test, respectively. Without pre-loading, supporting structures did not influence the fracture load or failure mode. Partial fractures were the most common failure mode. Pre-loading promoted the severity of the failure mode, although the fracture load among the framework designs was not influenced. In the AS group, prefailures were observed during pre-loading, and complete fractures were significantly increased after pre-loading. In contrast, the failure mode of the BLS group remained unchanged, showing only partial fracture even after pre-loading. This Ce-TZP/A framework design, comprised of an anatomical shape with additional buccal and lingual structures, has the potential to reduce the chipping of the veneering porcelain.

## 1. Introduction

The development of dental computer-aided design/computer-aided manufacturing (CAD/CAM) has increased zirconia use in all-ceramic restorations. Zirconia-based all-ceramic restorations have superseded the porcelain-fused-to-metal-ceramic restorations as the framework of crowns and fixed partial dentures (FPDs) for esthetic and biocompatibility reasons [1]. The success rate of zirconia-based all-ceramic restorations is adequate and comparable to that of metal-ceramic restorations [2,3]. However, technical complications, such as chipping of porcelain veneer on bilayered porcelain/zirconia ceramics, can occur [4]. It has been reported that zirconia-based all-ceramic restorations revealed a high rate of veneering fracture, ranging from 6% to 15% over a three- to five-year period, while the veneering fracture rate for metal-ceramic restorations ranges from 4% to 10% over 10 years [5]. In particular, the frequency of chipping has been reported to be higher in zirconia FPDs than in those comprising metal-ceramic FPDs [6]. Causes of chipping are not clearly understood [7]; however, various factors such as the zirconia framework thickness, porcelain veneering method and firing procedure affected the chipping behavior [8,9,10,11].

Framework modification is one of the most important factors for reducing technical complications [12]. The conventional uniform-thickness design of zirconia copings has been modified to an anatomical framework design to support the occlusal cusps [13]. Consequently, the porcelain veneer attained an even thickness. This anatomical framework design improved the fracture strength and fatigue reliability, and also reduced the chipping area of yttria-stabilized tetragonal zirconia polycrystal (Y-TZP) crowns [14,15]. After that, the anatomical framework design was further modified. A lingual supporting structure was added by extending the height in the lingual cervical margin and thickness in the proximal area was increased to reduce the amount of porcelain veneer in the non-visible area [16]. This lingual supporting structure has been clinically tested [17].

Y-TZP is commonly used as the framework of dental prostheses. Because of its superior mechanical properties compared with other ceramics, fractures of Y-TZP frameworks are rare incidents [18,19]. One risk of Y-TZP is its susceptibility to structural transformation from the tetragonal to the monoclinic phase under hydrothermal conditions; this structural transformation is accelerated in the human body [20]. In an *in vitro* study, it has been demonstrated that Y-TZP dental ceramics are also affected by this low-temperature degradation (LTD), depending on the grain size, residual stress, and stabilizer type and contents [21]. In another study, long-term use of Y-TZP is considered to induce LTD [22]. However, the clinical performance of Y-TZP-based prostheses showed favorable results [23,24]. Most of these studies used framework designs with anatomical shapes. Unfortunately, the anatomical shape was insufficient to eliminate chipping completely in clinical practice [25]. Moreover, after veneering with porcelain, a lingual supporting structure seems to be particularly affected by LTD because its framework is exposed to wet conditions in the oral cavity, whereas an anatomical framework design is not exposed. In an *in vitro* study, a lingual supporting structure did not improve the fatigue resistance of Y-TZP [26].

As an alternative to Y-TZP, ceria-stabilized tetragonal zirconia/alumina nanocomposite (Ce-TZP/A) was recently proposed as a novel framework material in dentistry [27,28]. Ce-TZP/A features superior fracture toughness and is not affected by LTD [29,30]. These improved material properties may also increase resistance to chipping in the porcelain veneer. Moreover, Ce-TZP/A frameworks exhibit superior fracture strength compared to Y-TZP [8], thus permitting a reduction of framework thickness. In a short-term clinical observation, Ce-TZP/A-based FPDs with a lingual supporting structure showed sufficient stability [31]. However, even if the lingual supporting structure withstands occlusal forces in the lingual side, the buccal cusp which has no corresponding support is likely to develop chipping. Thus, an additional supporting structure is necessary to reduce chipping in the buccal cusp.

In this study, a novel approach comprising supporting structures for the buccal and lingual cusps was adopted for Ce-TZP/A-based all-ceramic restorations. For esthetic reasons, the buccal supporting structure was fully covered by veneering porcelain. Evidence on long-term clinical use and the influence of framework design is scarce. The aim of the present study was to examine the fracture load and failure mode in all-ceramic crowns with different Ce-TZP/A framework designs. The null hypotheses were that the addition of buccal and/or lingual supporting structures to a Ce-TZP/A framework would not improve either the fracture load or failure mode.

## 2. Materials and Methods

### 2.1. Preparation of Ce-TZP/A-Based All-Ceramic Crowns

**Figure 1:**
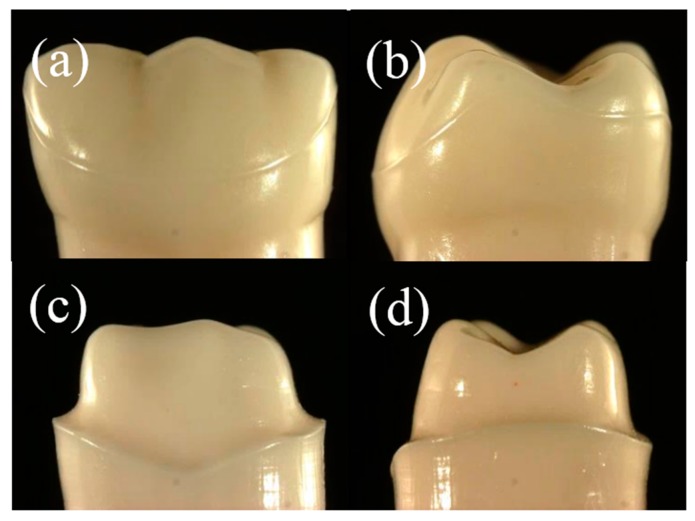
Photographs of the artificial tooth before tooth preparation (**a**,**b**) and after tooth preparation for all-ceramic restorations (**c**,**d**).

Four different Ce-TZP/A framework designs were evaluated. The frameworks were made from Ce-TZP/A blanks (C-Pro Nano-Zirconia, Panasonic Healthcare Co., Ltd., Tokyo, Japan) using a CAD/CAM system (C-Pro System, Panasonic Healthcare Co., Ltd.). First, the abutment tooth preparation was performed on an artificial left-mandibular first molar tooth (Simple Root Tooth Model A50-A-500, Nissin Products Inc., Kyoto, Japan) for an all-ceramic restoration in the standard manner, creating a 2 mm occlusal reduction of the functional cusp, a 1.5 mm occlusal reduction of the nonfunctional cusp, a 1 mm shoulder finish line with a rounded inner edge, and a convergence angle of approximately 6° (Figure 1). A metal tooth analog was replicated from the prepared artificial tooth by making a wax pattern and casting with cobalt-chrome (Co-Cr) alloy (StarLoy C, DeguDent GmbH, Hanau, Germany). Subsequently, the Co-Cr tooth analog was adjusted, polished, and scanned (D700-3SP Scanner, Panasonic Healthcare Co., Ltd.). After scanning, the zirconia frameworks were designed, milled, and sintered from Ce-TZP/A blanks according to manufacturer’s instructions.

**Figure 2:**
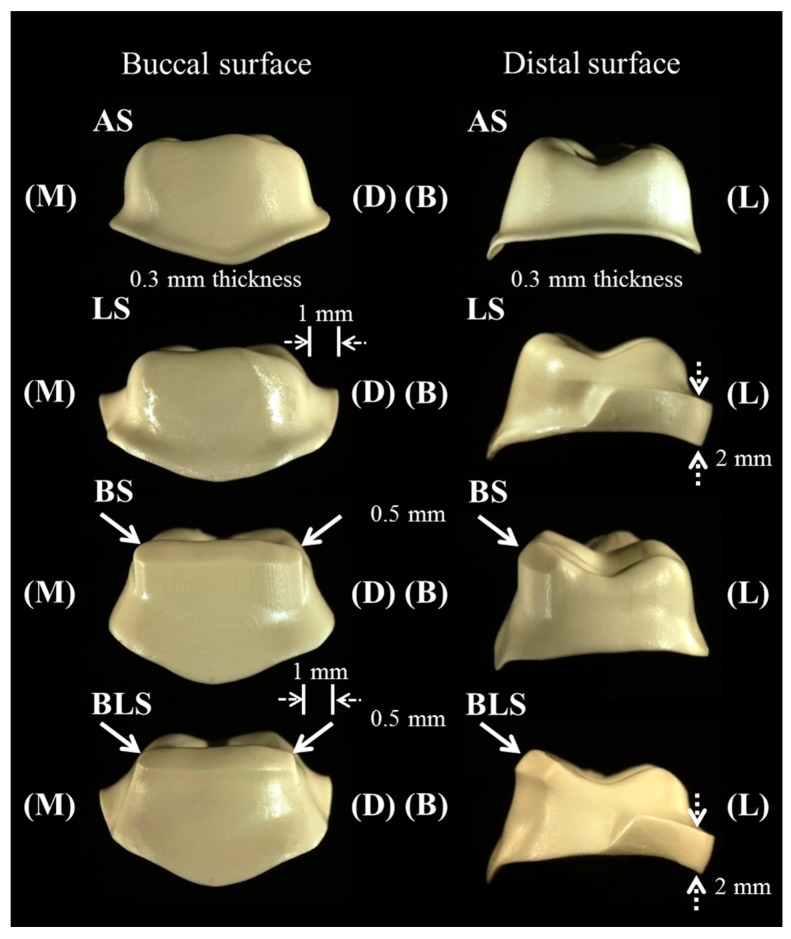
Ceria-stabilized tetragonal zirconia/alumina nanocomposite framework designs. White arrows and white dotted arrows show buccal and lingual supporting structures, respectively. Details of experimental groups are shown in Table 1. Abbreviations: B = buccal side; D = distal side; L = lingual side; and M = mesial side.

The four Ce-TZP/A framework designs were as follows (Table 1, Figure 2).

- AS: standard framework design; a 0.3 mm framework thickness with an occlusal anatomical shape (*n* = 24).
- LS: modified framework design; AS with a 0.7 mm framework thickness added to the lingual margin (lingual supporting structure). Total framework thickness including lingual supporting structure was 1.0 mm. Height of the lingual supporting structure was 2.0 mm (*n* = 24).
- BS: modified framework design; AS with a 0.2 mm framework thickness added at the external surface of the buccal cusp (buccal supporting structure). Total framework thickness including the buccal supporting structure was 0.5 mm (*n* = 24).
- BLS: modified framework design; AS with additional buccal and lingual supporting structures (*n* = 24).

After sintering, the fitness of each framework was examined and corrected if necessary. Each sintered framework was veneered with feldspathic ceramic via the layering technique used to fabricate all-ceramic crowns. Before veneering, the surface of the framework was blasted with 50 μm Al_2_O_3_ particles at 0.2 MPa for 10 s. The distance between the framework surface and nozzle was 10 mm. All frameworks were cleaned with ethanol, followed by distilled water. The layering procedure comprised the following: wash-bake (VM 9 Effect Liner EL, VITA Zahnfabrik H. Rauter GmbH & Co. KG, Bad Säckingen, Germany); first and second dentin (VM 9 Base Dentin A3, VITA Zahnfabrik H. Rauter GmbH & Co. KG); and glazing, which was performed in a dental furnace (Austromat 624, Dekema Dental-Keramiköfen GmbH, Freilassing, Germany) according to manufacturer’s instructions. Molds for layering porcelain on the Ce-TZP framework were made using an impression material (FUSION II Putty Type, GC Corp., Tokyo, Japan). In brief, a resin-up replica crown of each framework was made using self-curing acrylic resin (Palavit G, Heraeus Kulzer GmbH, Hanau, Germany). This replica crown was fixed on the Co-Cr tooth analog, and an impression was taken. For the dentin layer, base dentin powder was mixed with liquid and placed into the mold. Excess moisture was removed from the porcelain on the Ce-TZP/A framework using a tissue, and the framework was fired. Finally, glazing was performed.

Resin tooth analogs were made from cold polymerizing resin (Technovit 4000, Heraeus Kulzer GmbH), replicating the prepared artificial tooth. The inner surfaces of the crowns and the surfaces of the resin tooth analogs were sandblasted and ultrasonically cleaned. After pretreatment, Ce-TZP/A-based crowns (*n* = 96) were cemented to resin tooth analogs using self-adhesive resin cement (RelyX Unicem 2, 3M Deutschland GmbH, Neuss, Germany). This dual-cure cement was cured by chemical polymerization. During the cementation process, a constant load of 10 N was applied at the central fossa of each specimen, using a custom-made loading device and a stainless steel ball (diameter 4 mm). Excess cement was removed during curing. After cementation, the marginal gap of each specimen was confirmed to be in the range of clinical acceptance below 120 μm by stereomicroscopy (M400 Photomicroscope, Wild Heerbrugg AG, Heerbrugg, Switzerland) [32]. Cemented specimens were embedded in acrylic resin blocks (Palavit G, Heraeus Kulzer GmbH) in such a way that the long axis of the tooth was 2 mm below the margin line and stored in distilled water for 24 h at 37 °C. Prior to fracture loading test, half of the specimens in each experimental group (*n* = 12) underwent chewing simulation in form of mechanical pre-loading under wet conditions using a masticator (Willytec; SD Mechatronik GmbH, Feldkirchen-Westerham, Germany). Steatite balls (diameter 5 mm) were used as antagonists. The antagonist acted on the occlusal surface of each crown. The contact points of the antagonist were adjusted using red articulating paper to confirm vertical centric loading and three-point contact. The loading simulation, which was supposed to represent five years of oral service, used the following parameters [13]: weight, 49 N; cycles, 1.2 million; frequency, 1.4 Hz; and speed, 40 mm/s. After chewing simulation, the surface of each crown was observed for prefailure by stereomicroscopy.

### 2.2. Fracture Load

Fracture loading test of each crown either without mechanical pre-loading (−) or with mechanical pre-loading (+) was performed using a universal testing machine (Z010, Zwick GmbH, Ulm, Germany). The load was applied vertically at the central fossa of each crown using a stainless steel ball (diameter 4 mm) as a loading rod tip at a crosshead speed of 0.5 mm/min until fracture. After fracture, failure mode was observed by stereomicroscopy and scanning electron microscopy (SEM; LEO 1430, Carl Zeiss AG, Oberkochen, Germany). Failure mode was classified into two groups: partial fracture (cracking or chipping of porcelain veneer) and complete fracture (fracture of Ce-TZP/A framework or tooth analog).

### 2.3. Statistical Analysis

All data were examined for normal distribution by Shapiro-Wilk test and for equality of the variances by Levene test. The fracture load results were analyzed by two-way analysis of variance with framework design and mechanical pre-loading as independent factors followed by Tukey’s test for *post-hoc* comparisons (*α* = 0.05). For the purpose of statistical analysis, prefailure after mechanical pre-loading was considered as complete fracture. The failure modes’ results were analyzed by Fisher’s exact test. The statistical analyses were performed by the software packages Excel Statistics 2010 (Social Survey Research Information Co., Ltd., Tokyo, Japan) and R version 3.2.3 (The R Foundation for Statistical Computing, Vienna, Austria).

## 3. Results

### 3.1. Chewing Simulation as Mechanical Pre-Loading

Under stereomicroscopy observation, attrition marks due to contact with the antagonists were evident in all groups subjected to mechanical pre-loading. During chewing simulation, prefailure only occurred in the AS(+) group: three crowns (25%) exhibited cracking or chipping of the porcelain veneer and were excluded from subsequent fracture loading test.

### 3.2. Fracture Load

**Figure 3:**
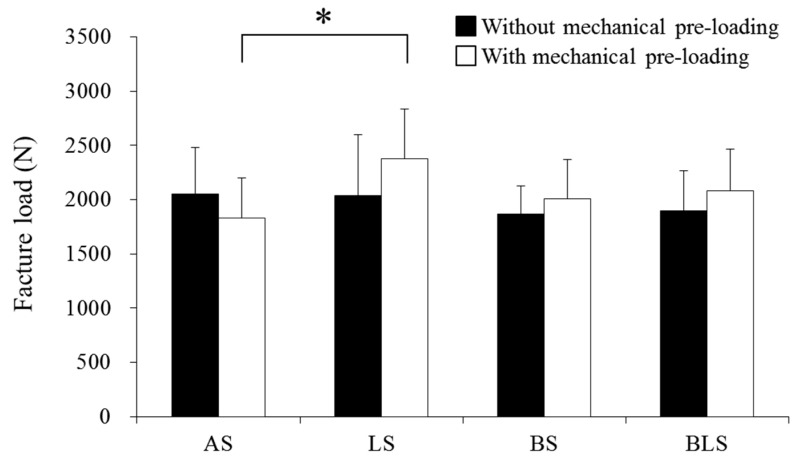
Fracture load of each experimental group. The asterisk indicates statistical significance of difference between groups (*p* < 0.05). Details of experimental groups are shown in Table 1.

The fracture load in each group is presented in Table 2; it ranged from 1866 ± 262 N (BS(−)) to 2049 ± 430 N (AS(−)) in the groups without mechanical pre-loading. No significant difference in fracture load was found between these groups. In the groups with mechanical pre-loading, the fracture load ranged from 1828 ± 374 N (AS(+)) to 2374 ± 464 N (LS(+)). The LS(+) group exhibited significantly higher fracture load than the AS(+) group (*p* = 0.0175); however, no significant difference was evident between the AS(+) and the BS(+) and BLS(+) groups. Mechanical pre-loading tended to increase mean fracture load in the LS(+), BS(+) and BLS(+) groups, while the fracture load of the anatomical design (AS(+)) was decreased, but the differences elicited by the pre-loading regime were not statistically significant (Figure 3).

### 3.3. Failure Mode

**Figure 4:**
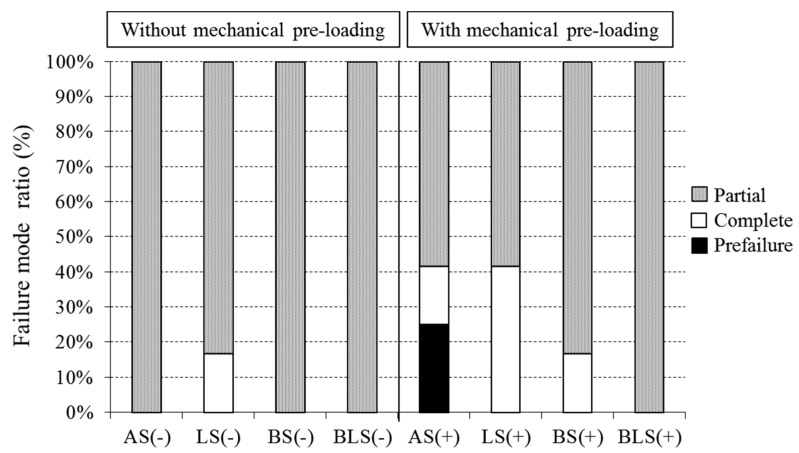
The distribution of failure mode ratio of each experimental group. Details of experimental groups are shown in Table 1.

The distributions of the failure mode ratios are presented in Figure 4. Partial fractures were the most common failure mode, irrespective of mechanical pre-loading. Without mechanical pre-loading, partial fracture ratios were 100% for the AS(−), BS(−), and BLS(−) groups and 83.3% for the LS(−) group. The remaining 16.7% in the LS(−) group were complete fractures. However, this difference between the LS(−) group and the other groups was not statistically significant.

After mechanical pre-loading, the failure mode shifted from partial to complete fracture. Complete fracture ratios ranged between 16.7% and 41.7% for the AS(+), LS(+), and BS(+) groups. Complete fracture of the AS(+) group (41.7%) was significantly enhanced compared to that of the AS(−) group (*p* = 0.0372). Only the BLS(+) group developed no complete fractures after mechanical pre-loading, and the partial fracture ratio remained at 100%. Complete fracture in this group was significantly lower compared to other groups (*p* = 0.0395).

**Figure 5:**
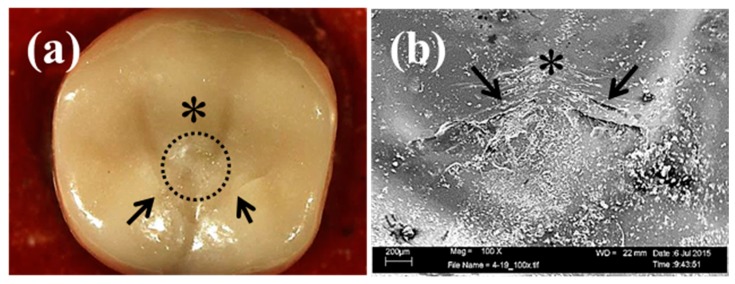

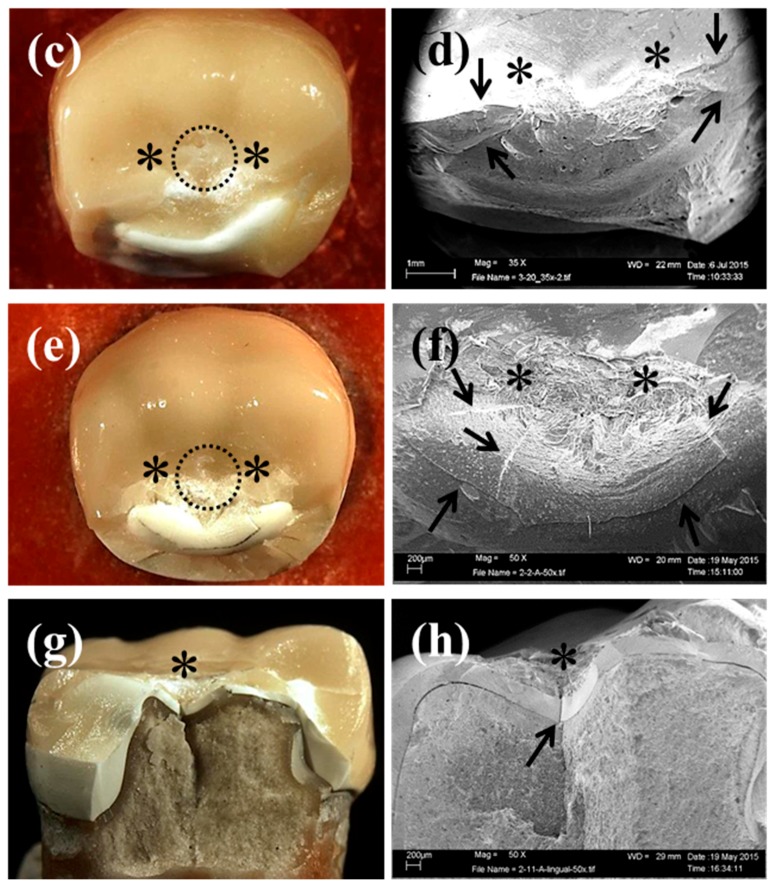
Microscopy (overview; left) and scanning electron microscopy (detail; right) observation of various failure modes. Cracking (**a**,**b**); chipping of porcelain (**c**,**d**); fracture of framework (**e**,**f**); and tooth analog (**g**,**h**). Black arrows, asterisks, and black dotted circles indicate fracture lines, load-bearing points, and areas, respectively.

**Figure 6:**
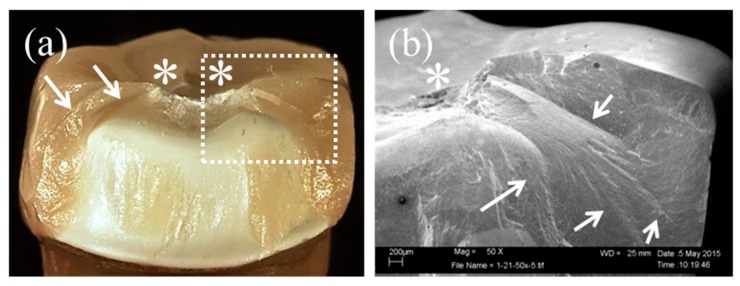

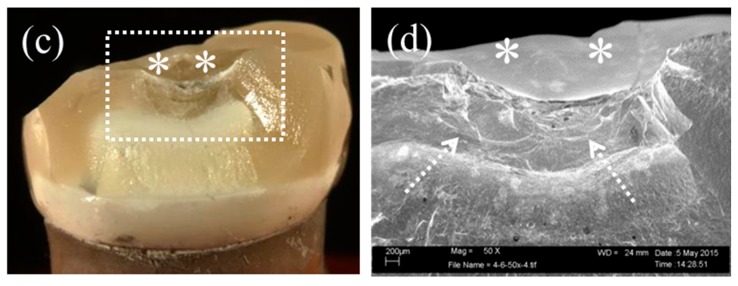
Lingual side views of microscopy (overview; left) and scanning electron microscopy (detail; right) observation; non-lingual supporting structure (**a**,**b**); lingual supporting structure (**c**,**d**). White arrows, white dotted arrows, and asterisks indicate fracture lines, arrest lines, and load-bearing points, respectively.

Details of each failure mode are presented in Table 3. One partial fracture type—cracking of the porcelain veneer—occurred in all frameworks except the BS(−) group. Two or three cases of cracking occurred in each group, and the cracked area was limited to the occlusal surface (Figure 5a,b). Chipping of the porcelain veneer, the major partial fracture, also occurred in all groups with varying incidence. The chipped area was mainly on the lingual side. The crack initiations originated at load-bearing points and areas (Figure 5c,d). In more than half of the cases, crack propagation then reached the lingual cervical margin in the groups without lingual supporting structures, such as the AS and BS groups (Figure 6a), and the interface of the lingual supporting structure in the groups with lingual supporting structures, such as the LS and BLS groups (Figure 6c). In the BLS group, the crack ratio on the buccal supporting structure was increased. SEM identified hackles and arrest lines. Complete fracture, *i.e.*, the fracture of the Ce-TZP/A framework, occurred rarely in the LS group between the mesio and distolingual cusps (Figure 5e,f). Furthermore, tooth analog fractures occurred in the AS(+), LS, and BS(+) groups (Figure 5g,h). The fractures originated from load-bearing points and propagated to the resin tooth analog (Figure 5h, black arrow).

## 4. Discussion

Chipping of veneered all-ceramic restorations is still a problem. One approach to overcome this problem is modification of the framework. The aim of this study was the investigation of the influence of different framework designs including several supporting structures on the fracture load and failure mode of veneered Ce-TZP/A crowns. A standard anatomical shape was compared to modified designs with supporting buccal/lingual structures. All groups were tested for fracture load and failure mode with or without a mechanical pre-loading regime simulating five years of clinical use. Mechanical pre-loading did not influence the fracture load in all groups. The failure mode, however, was affected by mechanical pre-loading in all groups except the BLS design. In the LS and BS groups, an increased amount of complete fractures was observed. In the AS group, even prefailure was observed after mechanical pre-loading. The BLS group, on the other hand, was the only design showing solely partial fractures under both tested conditions. Thus, our first null hypothesis was accepted, and our second was rejected.

Chipping commonly occurs at the cusp or marginal ridge area of molar all-ceramic crowns [25,33]. Veneering porcelain is a brittle material and requires support by the underlying framework. A lingual supporting structure improved the fracture strength not only in zirconia but also in glass-infiltrated alumina and metal-ceramic [34]. In the mandibular molar region, the buccal cusps act as functional cusps and are subjected to concentrated occlusal forces during chewing and biting [35]. An additional buccal cusp support is necessary to withstand those occlusal forces [12]. In this study, the concept of a framework design with additional buccal and lingual supporting structures was based on previous studies [17,36]. However, a supporting structure similar to the lingual supporting structure as described by Silva *et al.* [16] was not applicable to the buccal side as it would be directly visible and would not satisfy patients’ esthetic demands. An alternative framework design for metal-ceramic restorations features a two-sided supporting structure designed by adding buccal and lingual cusps to the framework’s surface; it exhibits improved fracture strength [36]. In this framework, the buccal supporting structure is invisible after veneering. The buccal and lingual supporting structures described in our study were selected for the simplicity of their manufacture. They incorporated features of both framework designs, comprising internal buccal and external lingual supporting structures after veneering with porcelain.

In this study, prefailure during mechanical pre-loading was regarded as a complete fracture for statistical analysis. The rationale behind this decision was that for the observed prefailure modes, the actual crown normally has to be removed and replaced by a new one in a clinical situation.

Without mechanical pre-loading, there was no significant difference in the fracture load and failure mode among the framework designs. Fracture load causes tensile stress in the porcelain veneer of bilayered all-ceramic crowns. The most frequent failure modes were partial fracture (*i.e.*, chipping of the porcelain veneer). Fracture originated at load-bearing points in all specimens. The chipped area was mainly on the lingual side, and crack propagation of the cohesive/adhesive fracture reached the cervical margin or interface of the lingual supporting structure (Figure 6a,c). Therefore, crack propagation from the central fossa towards the cervical margin and proximal area was identified by indicators such as hackles and arrest lines under SEM observation (Figure 6b,d). These findings may be explained by the location of load-bearing points. Kirsten *et al.* [37] used nine defined loading areas to simulate a physiological mastication behavior. The distribution of the maximum tensile stress was concentrated in the occlusal fissures between the mesiolingual and distobuccal cusps in Y-TZP crowns. In our study, the vertical load was applied at three contact points centered around the central fossa. Therefore, the load-bearing points were concentrated in the lingual side because two of the three contact points were located in the occlusal fissure between the mesio and distolingual cusps (Figure 5c).

Without mechanical pre-loading, partial fractures were observed in all groups, and only the LS(−) group showed already 16.7% of complete fracture. The different outcome compared to the BS(−) group may be explained by the location of the supporting structure. It has been reported that an extra-thick occlusal support reduced chipping and improved the fracture pattern [38]. Haker [36] suggested that additional two-sided supporting structures, which were similar to our buccal supporting structure, could enhance the porcelain’s strength by lowering the compressive stress between the porcelain and the metal framework due to stress dispersion. In our framework designs, the lingual supporting structure cannot increase the occlusal support area, while the buccal supporting structure can increase the area of the occlusal surface. Thus, only the buccal supporting structure could attain a stress dispersion. On the other hand, crack propagation could be stopped at the interface of the lingual supporting structure by its superior mechanical characteristic because the lingual supporting structure was only partially veneered with porcelain, whereas the buccal supporting structure was completely covered.

Generally, the influence of artificial aging on fracture load in Y-TZP crowns is investigated by a combination of thermocycling and cyclic loading [13,19]. The thermocycling treatment is included to simulate the influence of LTD on Y-TZP restorations under clinical conditions. In several studies, it has been reported that Ce-TZP/A exhibited complete resistance to LTD [39,40]. Therefore, in this study dealing with Ce-TZP/A crowns, only mechanical pre-loading was performed to simulate clinical use. After mechanical pre-loading, the fracture load tended to decrease in frameworks without additional supporting structures (AS), whereas fracture load tended to increase in frameworks with additional supporting structures (LS, BS, and BLS). These differences may be explained by two factors: zirconia transformation toughening and porcelain degradation.

It has been confirmed in several studies that zirconia phase transformation caused by external stress is one factor influencing fracture load. External stress, such as LTD, changed the phase transformation and produced positive as well as negative effects on Y-TZP mechanical properties [41]. The flexural strength increased by increasing the monoclinic contents up to 12% (transformation toughening); however, the flexural strength decreased with higher monoclinic contents. Von Steyern *et al.* [19] showed that Y-TZP crowns exhibited higher fracture loads after cyclic loading. They stated that the fracture load can be increased by cyclic loading up to a certain transformation toughening capacity. Our results indicate that the additional supporting structures seemed to have increased this transformation toughening capacity by increasing the framework sizes, thus contributing to the higher fracture loads in the LS, BS, and BLS groups. In contrast, the AS(+) group obviously had already reached the limit of its toughening capacity by the mechanical pre-loading stress. This assumption is confirmed by the high level of prefailures in the AS(+) group.

In our study, the incidence of complete fracture for all framework designs except for the BLS group was increased after mechanical pre-loading. It has been shown that even the combination of mechanical pre-loading and thermocycling does not affect the phase transformation or fracture properties of Ce-TZP/A [42]. However, the fatigue induced by cyclic loading can influence the strength of the porcelain veneer [43]. Obviously, mechanical pre-loading influenced the failure mode in our framework designs because continual compressive stress accelerated the degradation of the porcelain veneer and crack propagation. Particularly, prefailures of the AS group may have been caused by the more pronounced porcelain degradation in the thick porcelain layer of this group: In this framework design without supporting structures, a higher porcelain amount was required so that the ratio between the veneering ceramic and zirconia framework was suboptimal. It has been demonstrated that the amount or thickness of the veneering porcelain affects stress distribution within the porcelain layer in Y-TZP crowns [34,44]. The incidence of cracking rises with increasing the porcelain veneer thickness, at least for Y-TZP crowns [45]. In our study, the ratio of porcelain to Ce-TZP/A framework (1.2–1.7 mm/0.3 mm = 4–5.7) is higher than in other studies using Y-TZP (1.0 mm/0.5–0.6 mm = 1.67–2) as base material [14,34].

The results of our tests indicate that a 0.3 mm framework without additional supporting structures offers insufficient support to an artificially aged, comparatively thick porcelain layer under simulation of clinical conditions. Among the supporting structure designs, the BLS design can reduce the amount of porcelain veneer compared to the BS and LS group so that this framework design showed significantly lower complete fractures after mechanical pre-loading. Although the framework design of the BLS group consists of a more complicated structure than the other tested designs, this framework combines the advantages of both buccal and lingual supporting structures.

## 5. Conclusions

The modification of anatomically designed Ce-TZP/A crowns by the addition of buccal and/or lingual supporting structures (BS, LS and BLS) tended to increase the fracture load after mechanical pre-loading simulating five years of clinical use for all three tested designs. The increase in the fracture load, however, was statistically significant only for the lingual support group LS(+). Incidence and severity of the failure mode, however, were positively affected by all framework designs with additional supporting structures. Without mechanical pre-loading, the most common failure modes were partial fractures among all framework designs. After mechanical pre-loading, 41.7% complete failures were observed in the anatomical design (AS) reference group, partly the samples that already failed during simulated clinical use (pre-failure). All modified designs with buccal and/or lingual supporting structures led to a more or less pronounced reduction of prefailures and complete failures, shifting the failure mode from complete to partial fracture.

Especially one Ce-TZP/A framework design (BLS), comprised of a basic anatomical shape modified with additional buccal and lingual structures, prevented total fractures completely, showing only partial fractures even after mechanical pre-loading. Within the limits of this *in vitro* study, this Ce-TZP/A framework design has shown potential to reduce chipping of the veneering porcelain in clinical practice.

## Figures and Tables

**Table 1:** Experimental groups in this study.

Experimental Group	Mechanical Pre-Loading	Code	*n*
Occlusal anatomical shape: AS	Without (−)	AS(−)	12
	With (+)	AS(+)	12
AS with an additional lingual supportingstructure: LS	Without (−)	LS(−)	12
	With (+)	LS(+)	12
AS with an additional buccal supporting structure: BS	Without (−)	BS(−)	12
	With (+)	BS(+)	12
AS with additional buccal and lingual supporting structures: BLS	Without (−)	BLS(−)	12
	With (+)	BLS(+)	12

**Table 2:** Fracture loads (N) of all-ceramic crowns using different Ce-TZP/A framework designs (Mean ± S.D.)

Experimental Group	AS	LS	BS	BLS
Without mechanical pre-loading (−)	2049 ± 430 ^A,a^	2040 ± 562 ^A,a^	1866 ± 262 ^A,a^	1897 ± 371 ^A,a^
With mechanical pre-loading (+)	1828 ± 374 ^A,a^	2374 ± 464 ^A,b^	2009 ± 360 ^A,ab^	2081 ± 387 ^A,ab^

Results of statistical analysis are represented by upper and lower case letters. Different uppercase letters in the same column indicate that the groups are significantly different (*p* < 0.05). Different lowercase letters in the same row indicate that the groups are significantly different (*p* < 0.05). Details of experimental groups are shown in Table 1.

**Table 3:** Number of respective failure modes of all-ceramic crowns using different Ce-TZP/A framework designs.

Experimental Group	Prefailure		Partial Fracture		Complete Fracture	
	Cracking of Porcelain	Chipping of Porcelain	Cracking of Porcelain	Chipping of Porcelain	Cracking of Porcelain	Chipping of Porcelain
AS(−)	-	-	3	9	0	0
AS(+)	2	1	2	5	0	2
LS(−)	-	-	3	7	1	1
LS(+)	0	0	2	5	1	4
BS(−)	-	-	0	12	0	0
BS(+)	0	0	2	8	0	2
BLS(−)	-	-	2	10	0	0
BLS(+)	0	0	2	10	0	0

Details of experimental groups are shown in Table 1.
